# Evaluation of scFv protein recovery from *E. coli* by in vitro refolding and mild solubilization process

**DOI:** 10.1186/s12934-019-1053-9

**Published:** 2019-01-14

**Authors:** Animesh Sarker, Abhishek Singh Rathore, Rinkoo Devi Gupta

**Affiliations:** Faculty of Life Sciences and Biotechnology, South Asian University, New Delhi, India

**Keywords:** Single chain variable fragment, Inclusion bodies, Refolding, Mild solubilization, Bacterial protein production

## Abstract

**Background:**

The production of therapeutically active single chain variable fragment (scFv) antibody is still challenging in *E. coli* due to the aggregation propensity of recombinant protein into inclusion bodies (IBs). However, recent advancement of biotechnology has shown substantial recovery of bioactive protein from such insoluble IBs by solubilization and refolding processes. In addition, gene fusion technology has also widely been used to improve the soluble protein production using *E. coli.* This study demonstrates that mild-solubilization and in vitro refolding strategies, both are capable to recover soluble scFv protein from bacterial IBs, although the degree of success is greatly influenced by different fusion tags with the target protein.

**Results:**

It was observed that the most commonly used fusion tag, i.e., maltose binding protein (MBP) was not only influenced the cytoplasmic expression in *E. coli* but also greatly improved the in vitro refolding yield of scFv protein. On the other hand, mild solubilization process potentially could recover soluble and functional scFv protein from non-classical IBs without assistance of any fusion tag and in vitro refolding step. The recovery yield achieved by mild solubilization process was also found higher than denaturation–refolding method except while scFv was refolded in fusion with MBP tag. Concomitantly, it was also observed that the soluble protein achieved by mild solubilization process was better structured and functionally more active than the one achieved by in vitro refolding method in the absence of MBP tag or refolding enhancer.

**Conclusions:**

Maltose binding protein tagged scFv has shown better refolding and solubility yields as compare to mild solubilization process. However, in terms of cost, time and tag free nature, mild solubilization method for scFv recovery from bacterial IBs is considerable for therapeutic application and further structural studies.

**Electronic supplementary material:**

The online version of this article (10.1186/s12934-019-1053-9) contains supplementary material, which is available to authorized users.

## Background

In the recent time, antibody based biomolecules are being frequently used in disease diagnosis and prevention. One of such widely used biomolecules is single chain variable fragment (scFv) antibody which is particularly attractive due to its smaller size, low immunogenicity and low cost production [1]. However, production of fully functional scFv antibody protein from bacteria is still challenging due to its misfolding and formation of inclusion bodies (IBs) [2, 3]. Excess production of recombinant protein in the bacterial cytosol often triggers the partially folded proteins to interact with each other that results in protein aggregation thus IB formation [4, 5]. The reducing environment of the bacterial cytoplasm also contributes to protein misfolding as well as IB formation by inhibiting intra-disulfide bond formation [6]. Generally, IBs are protein aggregates with very little or no biological activity [7]. Currently, with the aid of recombinant technology and protein engineering, several strategies such as gene fusion technology, co-production of chaperones and foldases, use of mutated host strains, lowering expression temperatures and inducer concentration have been exploited for efficient soluble protein production in *E. coli* [8, 9]. Now a days, denaturation and refolding of classical and non-classical IBs are becoming prevalent to recover soluble and active protein from *E. coli* [10–12]. In this study, gene fusion technology was applied along with denaturation–refolding process to recover soluble scFv protein from bacterial IBs. In addition, mild solubilization strategies have been verified as high yielding and cost effective in comparison to the complete denaturation and in vitro refolding technique [13]. To obtain bioactive protein, IBs are usually denatured with high concentration of chaotropes such as urea or guanidine hydrochloride (GdnHCl) [14]. Sometimes, β-mercaptoethanol and dithiothreitol (DTT) are also added for proteins with multiple cysteine residues to reduce incorrect disulfide bonds [15]. High concentration of chaotropic agent results in complete denaturation of insoluble IBs which are further subjected to a single refolding step to recover soluble protein [16]. Alternatively, soluble protein can also be recovered directly from non-classical IBs by using non-denaturing solubilizing agents such as *N*-lauroyl sarcosine, dimethylsulfoxide (DMSO) and low percentage (~ 5%) of *n*-propanol [7, 17, 18]. Several studies have mentioned earlier that the recovery yield of soluble protein from bacterial IBs depends on its nature and the strength of denaturing agents which are applied during the solubilization process [7, 19].

Generally, in vitro refolding technology includes complete denaturation of classical IBs with high concentration of denaturing agents followed by dilution in refolding buffer and subsequent dialysis to remove excess salts and other denaturing agents [2, 20, 21]. The major drawbacks of this method are its complex and expensive operational process which further needs extensive optimization at a number of steps [22]. In addition, use of high concentration of denaturing agent results in complete denaturation of secondary structure of IB protein leading to re-aggregation during successive refolding process [23]. Consequently, recovery yield of soluble protein is greatly reduced during the process of in vitro refolding. Currently, with the knowledge of genetic and protein engineering, novel tailored-made strategies have been applied to improve the soluble protein production from *E. coli.* Gene fusion is one such commonly used technology that has shown to improve the soluble protein production in bacteria [24]. A widely accepted study has shown that fusion of maltose binding protein (MBP) at the C-terminal of recombinant scFv greatly influences its soluble and functional expression in *E. coli* [25, 26]. Another recent study has shown that MBP-scFv conjugates retain better in vitro solubility and stability as compared to untagged scFv [26]. The current study has also shown that MBP tag not only influences the cytoplasmic expression of *E. coli,* but also greatly improves the in vitro refolding yield of scFv protein. On the other hand, the mild solubilization process using high pH buffers, high pressure, detergents, organic solvents and low concentration of chaotropes help to retain native secondary structure of the protein [23, 27–30].

## Results

### Construction of scFv antibody

Generally, scFv antibody is constructed with variable heavy (V_H_) and variable light (V_L_) chain by covalently linking with a short peptide that is mostly composed of Glycine, Serine and Alanine residues [31]. Herein, the amino acid sequences of all the six CDRs of variable heavy chain (V_H_) and variable light chain (V_L_) were assembled from an anti-fusion loop dengue E53 Fab antibody (PDB: 3IXY) by joining with 15 amino acid (Gly_4_Ser)_3_ long linker to create a full length scFv antibody protein molecule [32]. The structural integrity of newly designed scFv molecule was verified by in silico homology modelling and superimposition on parental E53 Fab molecule (Additional file 1: Figure S1). After codon optimization for *P. pastoris and E. coli,* the newly designed scFv gene was de novo synthesized from GeneArt (Invitrogen™).

### Cloning of scFv antibody gene

The scFv insert gene was restriction digested from recombinant pJET1.2 cloning vector and was further used for recombination in pET28a(+), pGEX-4T-1 and pMAL-p5X expression vector. The resulting recombinant plasmids, i.e., scFv-pET28a(+), His.scFv-pET28a(+), GST.scFv-pGEX-4T-1 and MBP.scFv-pMAL-p5X (Additional file 2: Figure S2, Additional file 3: Figure S3) were further subjected to restriction digestion with the corresponding set of restriction enzymes in order to confirm the proper ligation. In agarose gel electrophoresis, all of the recombinants showed fall out at around 700 bp DNA ladder that confirmed proper integration of the desire scFv insert of 732 bp length in the target expression vector.

### Expression of scFv antibody protein

ScFv antibody proteins without any tag and with different fusion tags such as 6xHis, GST and MBP, were expressed in *E. coli* in different growth condition. In normal expression condition, none of the verified scFvs, neither with 6xHis, GST and MBP tags nor without any tag were expressed in soluble form. The scFv expression in *E. coli* was also analyzed by lowering expression temperature and inducer concentrations. Unfortunately, the scFv expression in soluble fraction was remaining almost unchanged as it was in *E. coli* under normal expression condition (Additional file 4: Figure S4). However, due to the reducing condition of *E. coli* cytoplasm, expressed scFvs are hardly capable to form disulfide bonds, hence mostly renders in insoluble aggregation as IBs [25, 33, 34]. The most common solution for that is secretion of the scFv to the bacterial periplasm where the oxidizing condition facilitates the disulfide bonds formation [35]. It has already been reported that scFv in fusion with MBP, efficiently expresses in bacterial periplasm as a soluble and active form [26]. In addition, MBP is a stable monomer and does not induce artificial dimerization or aggregation [36]. Here, a significant level of scFv expression in soluble fraction was also observed (Additional file 5: Figure S5) while scFv was expressed in fusion with MBP tag and was extracted following periplasm specific protocol. Several biochemical and biophysical studies have revealed that MBP carries large hydrophobic cleft exposed on its surface where fused polypeptide interacts with fusion partner rendering it inaccessible to form aggregation [36, 37]. Moreover, MBP have certain structural flexibility regarding to the hydrophobic cleft on its surface where different polypeptide can accommodate easily and block self-association during folding process.

### Mild-solubilization, denaturation and refolding of scFv inclusion bodies

**Fig. 1:**
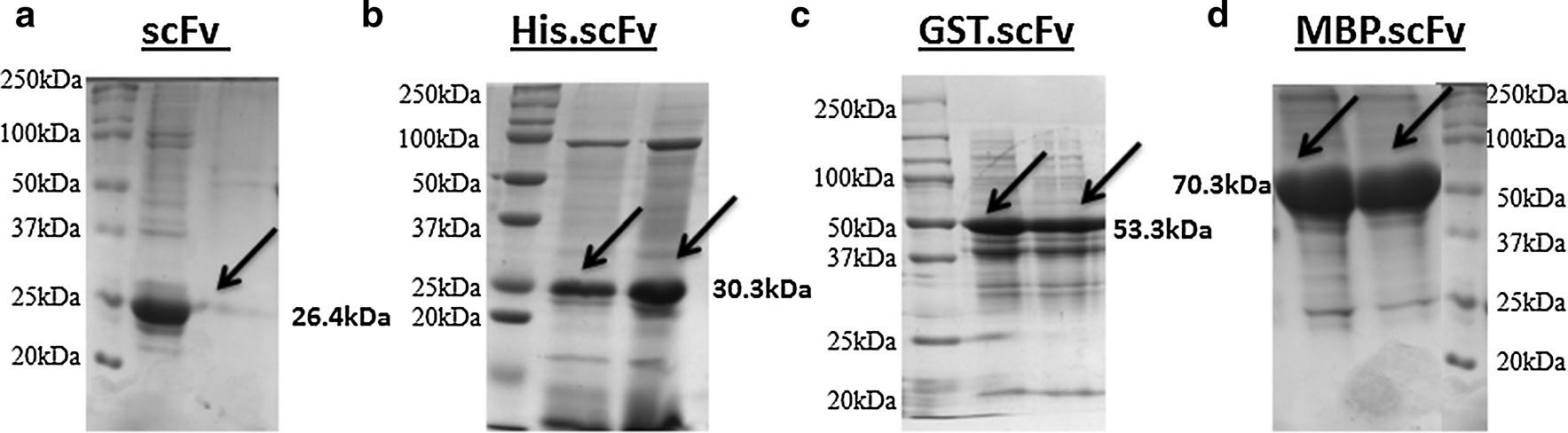
Band pattern of soluble scFv proteins on SDS-PAGE achieved by mild solubilization and denaturation–refolding method. **a** The supernatant of tag free scFv-pET28a(+) pellet, recovered by mild-solubilization process. The soluble fractions of **b** His-scFv, **c** GST-scFv and **d** MBP-scFv pellets, recovered by denaturation–refolding method

ScFvs in fusion with three different tags such as 6xHis, GST and MBP were expressed in fixed 500 mL bacterial culture; classical IBs were isolated and solubilized with strong chaotropes such as 6 M GdnHCl and 10 mM DTT. Entirely denatured and soluble scFvs were further allowed for refolding with buffer containing 5.0 M urea, 400 mM ArgHCl, 3.0 mM DTT, 4.0 mM GSSG and 100 mM Tris pH 8.1. Finally, high concentration of GdnHCl and other refolding salts were removed by slow dialysis. The soluble fractions of each concentrated and refolded scFv samples were then separated on SDS-PAGE. After staining the SDS gel with coomassie brilliant blue, prominent bands were observed in parallel with approximate molecular weight of corresponding scFvs that were fused with three different tags (Fig. 1). The scFv with MBP tag showed the most intense band, whereas GST-scFv showed least intense band compared to others. Therefore, evidently it is suggestive that MBP tag not only influences folding of its fusion partner during normal periplasmic expression, but also during in vitro refolding it augments proper folding. Several studies have also reported that MBP promotes solubility of fusion protein by showing its intrinsic chaperone activity, and it is more efficient when tagged at N-terminus rather than C-terminus [36, 38]. Conversely, GST is reported as poor solubility tag, always rendering its fusion partner in oligomer form as it carries four cysteine residues exposed to solvent that provide significant chances of oxidative aggregation [39]. Therefore it is plausible here that during refolding and concentration process, GST-scFv got aggregation rendering least amount of scFv in solution as it showed poor solubility in SDS-PAGE even in comparison to scFv fused with only 6xHis tag.

On the other hand, the recombinant scFv (without any tag) was also expressed in *E. coli* 500 mL LB culture at normal bacterial growth condition. Generally, in normal growth condition, major fraction of recombinant protein expresses in the form of non-classical IBs. Therefore, the resulting IBs were solubilized with mild denaturing agents such as 2 M urea, 5% DMSO, 5% isopropanol, 4 M GSSG in 50 mM PBS (pH 7.4). After high-speed centrifugation (greater than 20,000*g* for 10 min), the resulting supernatant was separated on 12% SDS polyacrylamide gel. Surprisingly, an intense band around 25 kDa protein marker was visualized in gel after staining with coomassie brilliant blue (Fig. 1a). The possible reason of increasing such prompt solubility yield comes from the fact that the mild solubilizing agent would not allow complete denaturation of protein, and might retain some extent of the existing secondary structure. Therefore, during re-naturation pathway, this partially folded protein does not allow more aggregation as like completely denatured protein that finally leads to improve the recovery yield of soluble scFv protein.

### Comparative analysis of solubility, refolding and overall pure scFv recovery yields achieved by mild-solubilization and in vitro refolding process

**Fig. 2:**
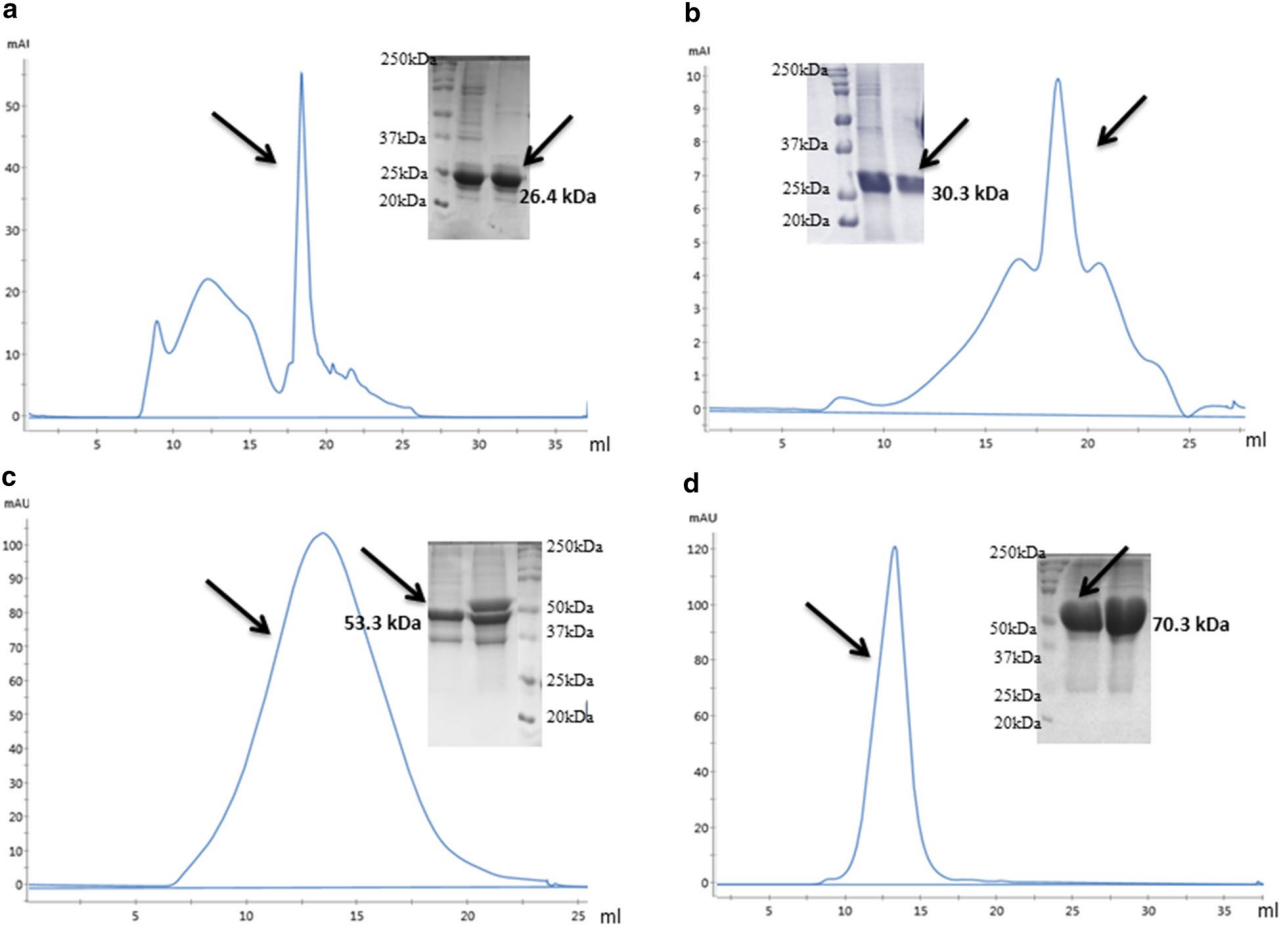
FPLC chromatogram and SDS-PAGE of purified scFv proteins without and with different fusion tags. **a** The chromatogram for tag free scFv protein solution achieved by using mild solubilizing agents and the pure scFv peak fraction was separated on SDS-PAGE. The chromatograms of **b** His-scFv, **c** GST-scFv and **d** MBP-scFv protein solutions achieved by complete denaturation followed by refolding-dialysis method and the pure peak fractions were also separated on SDS-PAGE

The soluble scFv proteins obtained from mild solubilization and denaturation–refolding method were purified using size exclusion chromatography. More than 95% purity level was attained for all of the scFv proteins except scFv with GST tag. The purity level of all scFvs with three different tags were checked and compared by using SDS-PAGE (Fig. 2). From the data of total protein content measured after IB solubilization, refolding and purification, the yields of solubility, refolding and overall pure scFv recoveries were calculated. It was noticed that the solubility yield (~ 46%) achieved by mild solubilization process (without following refolding step) was almost equal to the refolding yield (~ 49%) of scFv while it was fused with MBP solubility enhancer. Whereas, 6xHis and GST tags were not suitable enough to properly refold the scFv as it showed poor recovery yields around 11% and 7% respectively after following the in vitro refolding process (Table 1).

**Table 1:** Soluble and pure scFv protein yields recovered by mild solubilization and in vitro refolding methods

	Methods	Solublilized IB protein (mg)	Solubility and refolding yield (mg)	Solubility and refolding yield (%)	Soluble and pure protein yield (mg)	Recovery yield of pure scFv (%)
scFv	Mild solubilization	42.67 ± 6.0	19.84 ± 4.8	46.09 ± 4.3	12.3 ± 2.7	29.15 ± 1.8
His-scFv	Denaturation–refolding	65.33 ± 5.1	7.24 ± 1.3	11.08 ± 1.7	4.73 ± 0.5	7.26 ± 0.6
GST-scFv	Denaturation–refolding	72.0 ± 4.2	5.13 ± 0.8	7.12 ± 0.7	1.18 ± 0.4	1.67 ± 0.65
MBP-scFv	Denaturation–refolding	35.33 ± 5.4	17.42 ± 3.7	49.3 ± 4.8	15.59 × 3.4	44.13 ± 4.7

On the other hand, approximately 30% of pure scFv protein (without any tag) was recovered from insoluble IB pellet of *E. coli* by applying mild solubilizing method. However, in case of denaturation and refolding method, scFv with MBP tag was found to be the best in terms of recovery yield (~ 44%) whereas scFv in fusion with 6xHis and GST tag were identified as poor recovery potential around 7.2% and 1.6% respectively from insoluble IB pellet of *E. coli* (Table 1, Additional file 6: Figure S6). These findings are also symmetrical with our earlier remark that MBP enhances refolding yield by promoting solubility, and due to the aggregation propensity of GST tag, GST-scFv shows poor solubility [40] and refolding yield whereas, scFv with 6xHis tag showed little refolding potential as it has neither solubility capacity nor excess aggregation inclination [41]. Therefore, it can be inferred that without presence of solubility enhancing tag, scFv cannot refold properly in extra cellular condition.

### Structural analysis of soluble scFv protein

**Fig. 3:**
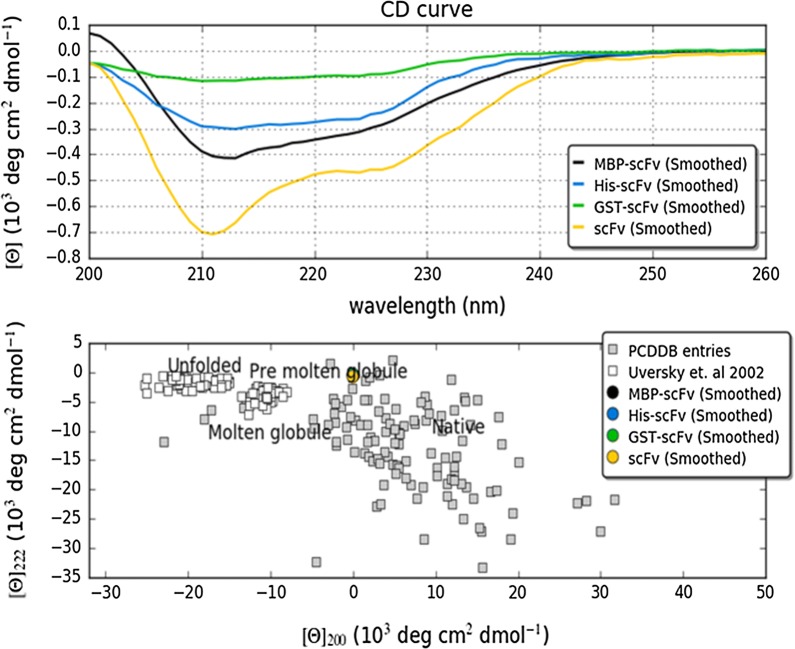
CD spectrum of scFv antibody protein with and without different fusion tags were recorded and analyzed with CAPITO. The graphical output for its area difference method is also shown. The best matching of reference dataset are scFv without any tag (orange) recovered by mild-solubilization method while scFvs achieved by denaturation–refolding method with different tags have not hit with reference data at all. Lower panel: the CD values at λ = 200 nm was plotted versus values λ = 222 nm to deduce the folding state of scFv in presence and absence of different tags

**Fig. 4:**
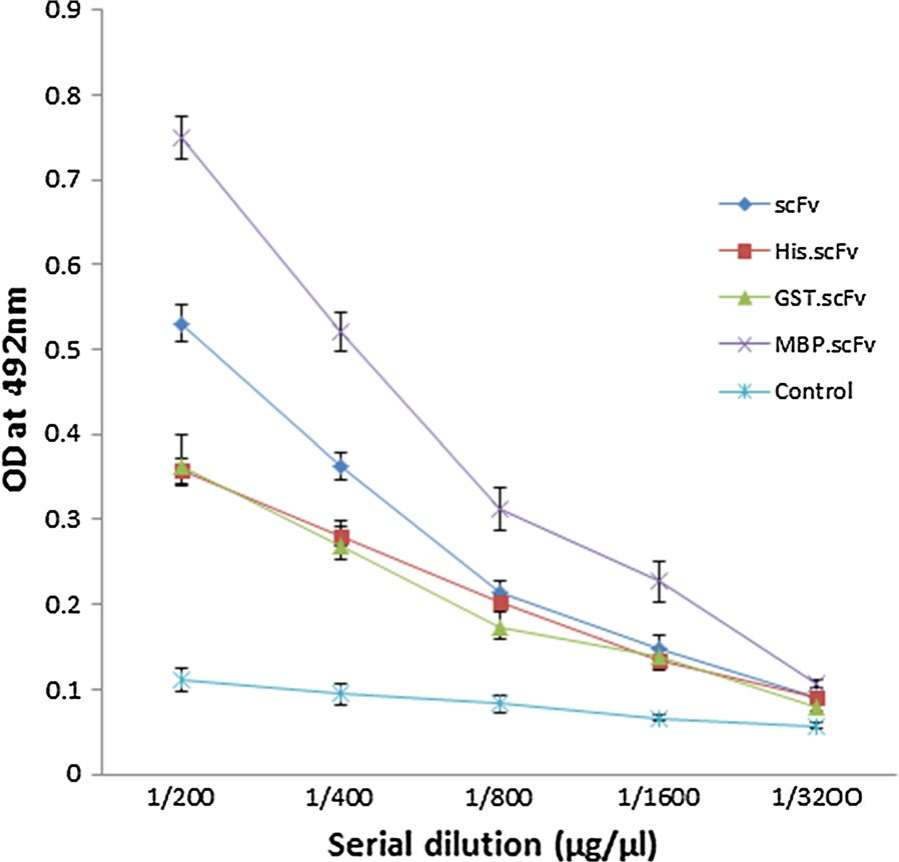
Direct ELISA of soluble scFv proteins and recombinant FuBC interaction. The recombinant FuBC protein was coated as 1 µg/µL concentration on each well of 96 well ELISA plate. Biotinylated scFv, His-scFv, GST-scFv and MBP-scFv were used as primary prey protein with initial concentration 5 µM followed by four serial double dilutions. PBST buffer without any biotinylated protein was used as primary for control sample

To understand the structural basis of soluble scFv protein achieved by mild solubilization and denaturation–refolding method, the effects of fusion tags on secondary structure were investigated by far UV CD spectrum. Originally, scFv (derived from PDB: 3IXY) is an exclusively β-sheet protein with at least 20 sheets. Unfolded protein which contains mainly irregular structural elements show a spectral minimum in the vicinity of 200 nm and an ellipticity close to zero in the vicinity of 222 nm [42]. The formation of secondary structures, either achieved by mild solubilization or denaturation–refolding methods, were characterized by negative bands at 208 nm and 222 nm wavelength. Furthermore, the ellipticity in case of scFv (without any tag) decreases significantly more at 208 nm and 222 nm that signifies better *β*-sheet formation as compared to scFvs with different tags (Fig. 3) [43]. In addition, the ‘double wavelength’ plot (λ = 222 nm versus λ = 200 nm) allowed clear visualization of the folding state of scFvs with and without different tags [44]. As shown in Fig. 4 (lower panel), it can be inferred that scFv achieved by mild solubilization retains the best folding state in absence of any fusion tag while in presence of different tags even MBP, the denaturation–refolding method was unable to retain soluble scFv at proper folding state.

### Binding analysis of soluble scFv proteins by ELISA

To study the binding activity of soluble scFv protein recovered by mild solubilization and denaturing–refolding method, direct ELISA and SPR analysis were performed. Here, the recombinant FuBC loop of dengue envelope protein has been used as bait since the amino acid sequences of V_H_ and V_L_ chains of our currently developed scFv molecule were taken from the anti-fusion loop of dengue E53 Fab antibody (PDB: 3IXY). It can also be noted here that Fu and BC are two highly conserved loop of dengue envelope domain II, were fused together to construct an ORF for the development of recombinant FuBC protein (Rathore et al. unpublished work). On the other hand, soluble scFvs achieved by mild solubilization (without any tag) and denaturation–refolding (with 6xHis, GST and MBP tags) methods were biotinylated and the binding was measured in direct ELISA by using them as primary antibody. Along with each set of experimental titer, one set of control titer was always used without applying any biotinylated scFv. The reading of multiple ELISA titers suggests that scFv with MBP fusion tag binds with dengue FuBC loop in higher intensity in compare to scFv with 6xHis and GST fusion tags. However, it should be noted that the signal achieved by MBP-scFv conjugate was not only developed by scFv alone but also mostly contributed by MBP tag as it shares 60% MW of total MBP-scFv fusion protein. On the other hand, soluble scFv recovered by mild solubilization method showed relatively higher titer value without assistance of any fusion tag (Fig. 4). It might be due to the result of using mild denaturing agent that does not denature protein completely, retains some extent of secondary structure, and showed better structural and functional integrity.

### Binding analysis of soluble scFv proteins by SPR assay

**Fig. 5:**
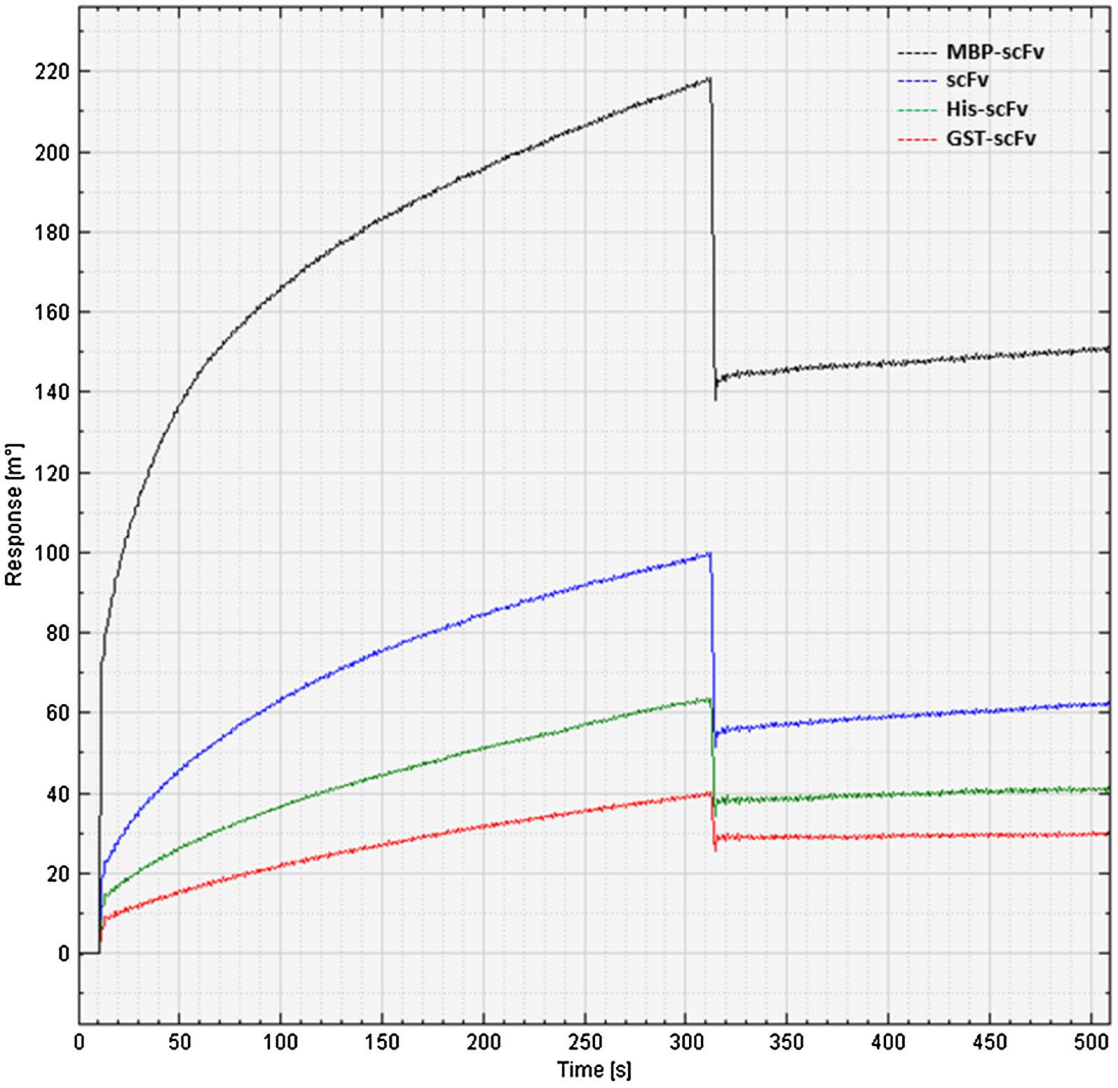
Sensorgram of soluble scFv proteins and recombinant FuBC loop interaction. The recombinant FuBC protein was immobilized on Biacore gold plate as bait. Refolded scFv proteins without any tag and with His, GST and MBP tags were used as analytes in mobile phase. Y axis is showing the refractive index as response of association and de-association of analytical substrate

SPR, a gold standard of quantifying bio-molecular interaction, was utilized here to validate scFv binding activity with recombinant FuBC loop of dengue envelope protein. Here also, recombinant FuBC loop of dengue envelope protein was immobilized on the thin gold plate of Biacore T200 instrument by amine coupling reaction, and all of the soluble scFvs either achieved by mild solubilization or in vitro refolding process were applied as analytes. It was observed that all of the soluble scFvs with and without different tags were capable to bind recombinant FuBC protein as all showed association–dissociation refractive index in SPR sensorgram. Similar to the ELISA results, MBP-scFv also showed highest binding response, whereas scFv (without any tag) achieved by mild solubilization process showed moderate binding activity; relatively higher refractive index compared to His-scFv and GST-scFv (Fig. 5). Since the refractive index depends on the total molecular mass of prey protein, the refractive index achieved by MBP-scFv conjugate must be contributed by MBP molecular mass as well.

## Discussion

Apparently, it is plausible that the in vitro refolding method in fusion with MBP tag is more effective for scFv recovery compare to mild solubilization process. However, scFv expressed in fusion with 6xHis and GST tags revealed poor recovery yield. Therefore, it is easily acceptable that scFv alone cannot refold properly and the yield achieved by in vitro refolding in fusion with MBP was due to solubility enhancing property of MBP. However, it is necessary to remove the fusion tag from final protein as it potentially interferes with proper structure and function of the target protein [45, 46]. Therefore, after removal of fusion tag, the final yield will at least be 60% less than of current recovery yield as the MBP (43 kDa) tag contributes more than 60% MW of the complete MBP-scFv (70.3 kDa) conjugate. In addition, several studies have also reported previously that tag removal is not only a time consuming expensive process but also greatly hinder overall recovery yield by acceleration of re-aggregation [43, 45]. On the other hand, mild solubilization process have shown around 30% overall scFv pure protein recovery yield without assistance of any solubility enhancing tag. It was also described previously that mild solubilization method uses non-classical IBs and low concentration of denaturing agents as it preserves native-like secondary structures during recovery process. Therefore, mild solubilization method is better for further functional and structural study or therapeutic applications with any recombinant protein as it is always preferred to work with protein comprising native conformation. In addition, mild solubilization would not require any refolding and tag removal process, as it would save extra-time, cost and potentially would avoid re-aggregation thus final recovery yield will be unchanged.

## Conclusions

Formation of IBs is the major challenge for large-scale recovery of bioactive protein from *E. coli*. Several gene fusions and refolding technology have been employed to improve the recovery yield of recombinant protein from bacterial IBs. Despite being widely utilized to improve soluble protein production in *E. coli*, fusion tags and refolding technology are still not well accepted due to non-native low yield protein recovery and re-aggregation propensity after removal of fusion tags. Therefore, the mild solubilization method could be utilized to recover bioactive protein from such insoluble IBs that provide a notable alternative to the conventional use of fusion tags (e.g., 6xHis, GST and MBP) and in vitro refolding process. The recovery yield achieved by the denaturation–refolding method was better than that achieved by the mild solubilization process while scFv was expressed and refolded infusion with MBP tag. Since, it is necessary to remove the tags before performing functional and structural analyses or any therapeutic applications as removing of MBP tag from MBP-scFv conjugates would decrease the overall yield by 60%, because MBP contributes more than 60% molecular weight of total MBP-scFv fusion protein. Hence, it can be concluded that both of the mild-solubilization and in vitro refolding strategies are capable to recover soluble scFv protein from bacterial IBs, however due to tag free nature and retaining native secondary conformation, the mild solubilization is comparatively better for recombinant protein production. Since it was observed that the soluble scFv protein recovered by the mild-solubilization process could retain proper structure and function without assistance of any fusion tag, it could be a better choice for efficient and economical recombinant protein production in *E. coli*. Further optimization of mild-solubilization method might result in the high-throughput recovery of therapeutic proteins from bacterial inclusion bodies.

## Materials and methods

### Chemicals and reagents

The principal components of bacterial culture: Luria–Bertani broth, tryptone and yeast extract; antibiotics: ampicillin, kanamycin; protein expression and analysis reagents: isopropyl β-d-1-thiogalactopyranoside (IPTG), Tris HCL and base, Glycine, lysozyme, phenylmethylsulfonyl fluoride (PMSF), sodium dodecyl sulphate (SDS), acrylamide/bis-acrylamide, ammonium persulfate (APS), tetramethylethylenediamine (TEMED), dithiothreitol (DTT), and ethylene-di-aminetetraacetic acid (EDTA), coomassie G-250 were purchased from Himedia and VWR life science. Inclusion body (IB) solubilizing and protein refolding reagents: guanidine hydrochloride (GdnHCl), urea, arginine hydrochloride (ArgHCl), isopropanol were from Sigma-Aldrich. PCR cloning kit: pJET1.2; all restriction enzymes; T_4_ DNA ligase and rapid protein assay BCA kit were from New England Biolabs (NEB) and Thermo Scientific. ELISA reagents: bovine serum albumin (BSA), Tween-20, Citric acid, H_2_O_2_, *o*-phenylenediamine dihydrochloride (OPD) were from Sigma-Aldrich and Alfa Aesar. Protein purification apparatus superose 12 10/300 size exclusion column was purchased from GE Healthcare. Affinity measuring Biacore T200 instrument and structure figuring Circular Dichroism (CD) Spectrometer facilities were provided by AIRF, Jawaharlal Nehru University, New Delhi-110021, India.

### Construction and synthesis of scFv antibody gene

The amino acid sequences of all the six CDRs of variable heavy chain (V_H_) and variable light chain (V_L_) of an anti-fusion loop dengue E53 Fab antibody (PDB: 3IXY) were joined by 15 amino acids (Gly_4_Ser)_3_ long linker to create a full length scFv antibody protein molecule. The structural integrity of the newly designed scFv molecule was verified by in silico homology modeling, and by superimposing on the parental E53 Fab molecule. After that, the corresponding scFv gene of the newly designed scFv antibody protein was de novo synthesized from GeneArt (Invitrogen™).

### Designing and cloning of scFv gene with and without fusion tag

Primarily, the synthetic scFv gene was PCR amplified with three pairs of primers (Additional file 7) that were flanked by three different sets of restriction sites. The PCR products of scFv were then recombined in the pJET1.2 PCR cloning vector, and digested with specific set of restriction enzymes to prepare targeted scFv inserts. The resulting scFv inserts were then eluted from the agarose gel and finally, sub-cloned into the pET28a(+) vector under the control of T7 promoter using *Nco*I and *Eco*RI restriction sites excluding N-terminal 6xHis tag sequence, and also in between *Eco*RI and *Xho*I restriction sites including 6xHis tag sequence at N-terminus (Additional file 2: Figure S2). Additionally, the scFv insert was also sub-cloned in pGEX-4T-1 vector using *Eco*RI and *Xho*I restriction sites, and in pMAL-p5X vector using *Nde*I and *Eco*RI restriction sites that included GST and MBP tags respectively at N terminus (Additional file 3: Figure S3). The recombinant constructs were further verified by Sanger sequencing.

### Expression and extraction of scFv recombinant protein

*Escherichia coli* BL21 (DE3) cells were transformed with the four recombinant vectors, namely scFv-pET28a(+), His.scFv-pET28a(+), GST.scFv-pGEX-4T-1 and MBP.scFv-pMAL-p5X that encloses only scFv gene (without any tags) and also scFv with 6xHis, GST and MBP tags respectively. The transformed cells were grown overnight in LB agar plates in presence of ampicillin for scFv-pET28a(+) and His.scFv-pET28a(+) clone and in presence of kanamycin for GST.scFv-pGEX-4T-1 and MBP.scFv-pMAL-p5X clone at 37 °C temperature. Single colony from each plate was then inoculated in four different LB broths with 100 µg/mL ampicillin or 50 µg/mL of kanamycin. The primary cultures grown overnight were re-inoculated in fresh LB broth with corresponding antibiotic, and followed to grown up at 37 °C till OD_600_ reached 0.5. All of the secondary cultures grown at 37 °C were then split into three groups, first group was induced with IPTG 1.0 mM concentration and grown at 37 °C for 4 h, second group was induced with 0.5 mM IPTG concentration and grown at 30 °C for 4 h and the third group was induced at 0.5 mM IPTG concentration but grown at 20 °C for overnight. The cells were then harvested by centrifugation at 4000*g* and the cell pellets were re-suspended with lysis buffer containing 50 mM Tris–HCl pH 8.0, 1 mM CaCl_2_, with 0.5% triton X-100, lysozyme 1.0 mg/mL, 1 mM EDTA and 1 mM PMSF. Cell lysates were kept on rocker for an hour at room temperature and then centrifuged at 12,000*g*. The crude supernatant was collected for all clonal expression, and the pellets were re-suspended with equal volume of lysis buffer without any lysozyme. Finally, scFv expression level in all supernatants and pellets were separated on SDS-PAGE.

### *Escherichia coli* expression and preparation of scFv inclusion bodies

The primary cultures of all scFv recombinant clones such as scFv-pET28a(+), His.scFv-pET28a(+), GST.scFv-pGEX-4T-1 and MBP.scFv-pMAL-p5X were used for inoculation of 0.5 L super-broth (10 g yeast extract, 16 g tryptone and 2.5 g NaCl) supplemented with 1 mM antibiotic, 4% MgSO_4_, and 20% sucrose. The secondary cultures were grown for 2–3 h at 37 °C up to OD_600_ 0.5, and to induce recombinant protein expression 1 mM IPTG was added. Subsequently, the cultures were incubated again for another 4 to 5 h at 37 °C, and the cells were harvested with centrifugation at 4000*g* for 20 min. Accordingly, the harvested cell pellets were re-suspended thoroughly in 60 mL TE 50/20 (Tris pH 8.0 50 mM and EDTA 20 mM) buffer and 0.5 mg/mL lysozyme, and cell suspensions were kept on rocker for 60 min at room temperature with periodic vigorous mixing. 10 mL of 5 M NaCl, 25% triton X-100 were added, and kept on rocker for another 30 min to stop the lysis reaction. Volume was made up to 100 mL with TE 50/20 buffer, and the lysed solution were allowed for sonication at 50% amplitude, 5 s on/off pulse with 1 min gap interval until sample thinned. After that, centrifugation was performed at 10,000*g* for 20 min at 4 °C, supernatant was removed, and the pellets were re-suspended with 50 mL TE 50/20 and 1% triton X-100. Similarly as before, four more times triton X-100 free sonication followed by centrifugation of re-suspended pellets was carried out to retrieve clean IBs. Before last centrifugation, two aliquots of 100 µL from total 100 mL of lysate were kept aside and later centrifuged separately at 14,000*g* for 5 min to check IB protein concentration and banding pattern on SDS-PAGE.

### Denaturation and mild-solubilization of scFv inclusion bodies

The pellets of scFv inclusion body prep retrieved from His.scFv-pET28a(+), GST.scFv-pGEX-4T-1 and MBP.scFv-pMAL-p5X clone were denatured thoroughly with 6 M GdnHCl, 100 mM Tris pH 8.1 buffer and 10 mM DTT at greater than 5 mg/mL approximate protein concentration (measured by BCA protein assay kit) which were then kept 2 h on rocker for gentle rotation. The dissolved proteins were then centrifuged at 25,000*g* for 20 min, and the supernatants were collected in 15 mL conical tubes for further refolding experiment. On the other hand, the non-classical IBs of tag free scFv-pET28a(+) clone was retrieved from *E. coli,* and was solubilized with mild denaturing agents such as 100 mM Tris pH 8.1, 2 M urea, 5% DMSO, 5% *n*-propanol and 4 mM oxidized glutathione; which was then allowed for overnight incubation at 4 °C temperature in gentle rotation. Finally, centrifugation was carried out at 20,000*g* for 20 min and the collected supernatant was checked on SDS-PAGE. The verified supernatant was further allowed for direct purification by size exclusion chromatography.

### Refolding and dialysis of solubilized scFv inclusion bodies

The solubilized proteins achieved from inclusion body denaturation, were added as 50 µg/mL concentrations in chilled buffer containing 4.5 M urea, 550 mM l-arginine HCl, 100 mM Tris–HCl pH 8.1, 1 mM of reduced glutathione (GSH), 0.1 mM of oxidized glutathione (GSSG), and were allowed for refolding reaction at 4 °C for 48 h. To remove excess urea and salts, the refolding protein solutions were transferred in membrane tube (10 kD MWCO), and were allowed for serial dialysis, first day in 2 L water for 200 mL refolding mixture in cold room (4 °C) followed by another 3 days in 2 L 10 mM Tris pH 8.1 buffer getting up to 10,000 times dilution. After 4 days, the diluted protein samples were concentrated by using Millipore-Amicon 15 mL filter (10 kD MWCO) up to concentration 0.1–1.0 mg/mL.

### Purification of soluble scFv antibody protein

The concentrated soluble scFv proteins either recovered by mild solubilization or denaturation–refolding methods were further allowed for FPLC (size exclusion chromatography) purification by using superose 12 10/300 column. The column was pre-equilibrated with PBS (50 mM phosphate buffer pH 7.4 and 150 mM NaCl), and the protein sample was also eluted with same PBS buffer. One mL protein sample was injected in each run by using 1 mL loop at 0.8 mL/min flow rate. The elution profile of the injected protein was monitored by UV absorbance at 280 nm on AKTA FPLC system with U9-L UV monitor. All of the peaks greater than 10 mAU were collected separately by using FPLC fraction collector, and further concentrated using 0.5 mL Millipore-Amicon filter. Pierce™ BCA protein assay kit (Pierce, USA) was used to determine the protein concentration of solubilized, refolded and purified form. All the protein samples were separated on 12% SDS-PAGE gel, and visualized by staining with coomassie-blue. Specific scFv bands were analyzed by comparing with pre-stained protein marker (PageRuler™).

### Structural analysis by CD spectroscopy

To study the structural integrity of scFv protein, the scFv proteins either achieved by mild-solubilized or in vitro refolding were characterized by CD spectroscopy. 400 µL of each scFv protein samples at 0.1 mg/mL concentration (diluted in buffer 10 mM Tris–HCl, pH 8.1) was taken in 1 mm cuvette for recording far UV spectra from 200 nm to 280 nm with step size of 1 nm to bandwidth 1 nm. The measurement was performed at room temperature (22 °C), and the UV spectra were recorded for each sample with five scan. Additionally, the CD spectra for each sample were presented by taking average of two independent measurements, each averaged of five scans.

### Binding analysis by direct ELISA

Binding analysis of mild-solubilized and refolded scFv antibody proteins was done by direct ELISA using 96 well flat bottom polystyrene plates. The recombinant FuBC loop of dengue envelope protein was coated on the ELISA plate, and biotinylated scFv without and with different tags were used as primary antibody followed by streptavidin–horseradish peroxidase conjugate as secondary antibody. Initially, the recombinant FuBC protein at 2 µg/mL concentration was diluted in 100 µL of 50 mM sodium carbonate buffer pH 9.5, and was coated into each well of 96-well ELISA plate for overnight. The unbound protein sample was washed with PBST buffer (PBS with 0.05% Tween 20), and the untenanted space was blocked with 2.5% (w/v) Bovine serum albumin (BSA) dissolved in PBS. To appraise the reaction rate, 100 µL of each biotinylated scFv without and with different tags were applied at different concentrations ranging from 5 µM to 300 nM and was incubated for 1 h at room temperature. Biotinylation of each scFvs was performed by using Thermo Scientific EZ-Link Sulfo-NHS-LC-Biotinylation Kit (Pierce High sensitivity Streptavidin-HRP), and by following the amine coupling protocol. Excess biotin was removed by using Zeba™ spin desalting columns after performing amine-coupling reaction. The unbound biotinylated scFvs during ELISA were washed with PBST buffer, and remaining plate was incubated with anti-goat HRP conjugated antibody diluted in PBS at 1:5000 ratios. After 30 min incubation at room temperature, the plate was washed three times with PBST, and later the direct ELISA signal was developed using *O*-phenylenediamine (OPD) substrate solution that was prepared in 100 mM sodium citrate buffer pH 5.0 with 0.003% H_2_O_2_. After 5 min of OPD substrate addition, 50 µL of 2 M H_2_SO_4_ was added to stop the colorimetric reaction. The intensity of developed color by the reactions were measured by using Biotek Synergy HT micro plate ELISA reader at 492 nm absorbance. It can be noted here that scFv was designed targeting FuBC loop of dengue envelope protein as its VH and VL domain sequences were driven from an anti-fusion loop dengue E53 Fab antibody (PDB: 3IXY).

### Validation of scFv binding by SPR assay

To validate the binding activity of scFvs either recovered by mild solubilization or by in vitro refolding were allowed for SPR interaction experiment on a Biacore T200 instrument (GE Healthcare). Recombinant FuBC protein was immobilized on gold plate of SPR device. For binding of FuBC protein sample on the surface of the gold disc, amine-coupling reaction was carried out. 1-Ethyl-3-(3-dimethylaminopropyl) carbodiimide (EDAC) and *N*-hydroxysuccinimide (NHS) were used for activation of the disc for coupling reaction. NHS activates the carboxymethyl groups by creating a highly reactive succinimide ester on disc surface, which reacts with amine, and other nucleophilic groups on proteins that subsequently helps binding of target protein. Ethanolamine was added to block the remaining activated carboxymethyl group. Then, all of the analytic scFv proteins at 100 µM concentration were injected in experimental flow cells, as well as a non-specific protein sample was injected in control flow cells to obtain its relative binding profile. In all of the SPR experiments, non-specific binding obtained in the control flow cell was subtracted from the refractive signal obtained in the experimental flow cell. For qualitative binding analysis, all of the scFvs (100 µM diluted in running buffer) were injected at a flow rate 20 µL/min over 2 min. In between injections, the surface of the sensor chip was regenerated by injecting 2 M NaCl for 15 s at the same flow rate. The bulk signal caused by refractive index differences between the flow buffer and the buffer containing the analytes were systematically excluded from the data-fitting process. Moreover, for FuBC surface coupling reaction, 150 mM NaCl, 3 mM EDTA, 0.005% surfactant P20, and 10 mM HEPES–NaOH, pH 7.4, were used, and 50 mM phosphate, 150 mM NaCl pH 7.1 was used as running buffer.

## Additional files

**Additional file 1: Figure S1.** Construction of scFv antibody gene. (**a**) The amino acid sequences of variable heavy chain (V_H_) and the variable light chain (V_L_) were joined with most commonly used peptide linker (G_4_S)_3_ sequence. Red and blue color residues are denoting CDRs of V_H_ and V_L_ respectively. (**b**) The newly designed scFv protein sequence was reverse translated in to DNA sequence by using computational tool. (**c**) Homology model of scFv protein molecule was designed by Swiss PDB viewer.

**Additional file 2: Figure S2.** Complete map of scFv-pET28a and His.scFv-pET28a plasmid. The scFv gene was cloned (**a**) using *Nco*I and *Eco*RI retriction sites of pET28a(+) to skip 6xHis tag and (**b**) using *Eco*RI and *Xho*I restriction sites of pET28a(+) to incorporate 6xHis tag during expression. The complete recombined map was created with SnapGene.

**Additional file 3: Figure S3.** Complete map of GST.scFv-pGEX-4T-1 and MBP.scFv-pMAL-p5X plasmid. The scFv gene was inserted (**a**) using *Eco*RI and *Xho*I restriction sites in pGEX-4T-1 expression vector and (**b**) using *Nde*I and *Eco*RI restriction sites in pMAL-p5X expression vector in order to express fusion scFv protein with GST and MBP tag respectively. The recombination was created with SnapGene.

**Additional file 4: Figure S4.** Expression pattern of scFv antibody protein without and with different fusion tags at different biological conditions. All of the four clones, scFv-pET28a(+), His.scFv-pET28a(+), GST.scFv-pGEX-4T-1 and MBP.scFv-pMAL-p5X were expressed in (**a**) 1 mM IPTG concentration at 37 °C for 4 h, then (**b**) 0.5 mM IPTG concentration at 30 °C for 4 h and (**c**) in 0.5 mM IPTG concentration at 20 °C for overnight.

**Additional file 5: Figure S5.** Periplasmic expression pattern of scFv antibody protein in fusion with MBP tag. MBP.scFv-pMAL-p5X recombinant plasmid was used to transform *E. coli* BL21 bacteria and cells were grown up to OD (A_600_ ~ 0.5) followed by expression with 0.3 mM IPTG concentration for 2 h at 37 °C. Harvested cells were used for protein extraction following periplasmic extraction method. The protein isolated from both in supernatant (S) and pellet (P) was used to separate by SDS 12 % polyacrylamide gel electrophoresis. Un-induced transformed cells were also extracted by periplasmic extraction method and also allowed for SDS 12 % polyacrylamide gel electrophoresis as control (C) sample.

**Additional file 6: Figure S6.** Percent (%) study of solubility, refolding and overall recovery yield of scFv protein. The IBs of scFv were solubilized mildly and strongly with the corresponding mild and strong denaturing agents. Completely denatured scFvs fusion with 6xHis, GST and MBP tags were further allowed for in vitro refolding. The recovery yields of soluble and refolded scFv proteins achieved by mild solubilization and denaturation-refolding method were converted into percent yields as per the quantity of initial pellet protein. Furthermore, overall percent yields of pure scFvs recovered by these two methods were calculated and compared with each other.

**Additional file 7.** Primer sequences for cloning scFv synthetic gene without and with His, GST and MBP fusion tags.

## References

[1] Yang X, Hu W, Li F, Xia H, Zhang Z (2005). Gene cloning, bacterial expression, in vitro refolding, and characterization of a single-chain Fv antibody against PreS1(21-47) fragment of HBsAg. Protein Expr Purif.

[2] Rudolph R, Lilie H (1996). In vitro folding of inclusion body proteins. FASEB J Off Publ Fed Am Soc Exp Biol.

[3] Burgess RR (2009). Refolding solubilized inclusion body proteins. Methods Enzymol.

[4] Carrió MM, Villaverde A (2005). Localization of chaperones DnaK and GroEL in bacterial inclusion bodies. J Bacteriol.

[5] Clark ED (2001). Protein refolding for industrial processes. Curr Opin Biotechnol.

[6] Singh SM, Panda AK (2005). Solubilization and refolding of bacterial inclusion body proteins. J Biosci Bioeng.

[7] Jevsevar S, Gaberc-Porekar V, Fonda I, Podobnik B, Grdadolnik J, Menart V (2005). Production of nonclassical inclusion bodies from which correctly folded protein can be extracted. Biotechnol Prog.

[8] Terpe K (2006). Overview of bacterial expression systems for heterologous protein production: from molecular and biochemical fundamentals to commercial systems. Appl Microbiol Biotechnol.

[9] Demain AL, Vaishnav P (2009). Production of recombinant proteins by microbes and higher organisms. Biotechnol Adv.

[10] Peternel S, Komel R (2010). Isolation of biologically active nanomaterial (inclusion bodies) from bacterial cells. Microb Cell Fact.

[11] Fursova KK, Laman AG, Melnik BS, Semisotnov GV, Kopylov PK, Kiseleva NV (2009). Refolding of scFv mini-antibodies using size-exclusion chromatography via arginine solution layer. J Chromatogr B Analyt Technol Biomed Life Sci..

[12] Tsumoto K, Shinoki K, Kondo H, Uchikawa M, Juji T, Kumagai I (1998). Highly efficient recovery of functional single-chain Fv fragments from inclusion bodies overexpressed in *Escherichia coli* by controlled introduction of oxidizing reagent–application to a human single-chain Fv fragment. J Immunol Methods.

[13] Upadhyay AK, Singh A, Mukherjee KJ, Panda AK (2014). Refolding and purification of recombinant l-asparaginase from inclusion bodies of *E. coli* into active tetrameric protein. Front Microbiol.

[14] Upadhyay V, Singh A, Jha D, Singh A, Panda AK (2016). Recovery of bioactive protein from bacterial inclusion bodies using trifluoroethanol as solubilization agent. Microb Cell Fact.

[15] Wingfield PT (2001). Use of protein folding reagents. Curr Protoc Protein Sci Editor Board John E Coligan Al.

[16] Yamaguchi H, Miyazaki M (2014). Refolding techniques for recovering biologically active recombinant proteins from inclusion bodies. Biomolecules..

[17] Park A-R, Jang S-W, Kim J-S, Park Y-G, Koo B-S, Lee H-C (2018). Efficient recovery of recombinant CRM197 expressed as inclusion bodies in *E. coli*. PLoS ONE.

[18] Tsumoto K, Abe R, Ejima D, Arakawa T (2010). Non-denaturing solubilization of inclusion bodies. Curr Pharm Biotechnol.

[19] Humer D, Spadiut O (2018). Wanted: more monitoring and control during inclusion body processing. World J Microbiol Biotechnol..

[20] Sun H, Wu GM, Chen YY, Tian Y, Yue YH, Zhang GL (2014). Expression, production, and renaturation of a functional single-chain variable antibody fragment (scFv) against human ICAM-1. Braz J Med Biol Res Rev Bras Pesqui Medicas E Biol..

[21] Ramón A, Señorale-Pose M, Marín M (2014). Inclusion bodies: not that bad….. Front Microbiol..

[22] Singh A, Upadhyay V, Panda AK, García-Fruitós E (2015). Solubilization and refolding of inclusion body proteins. Insoluble proteins: methods and protocols.

[23] Singh A, Upadhyay V, Upadhyay AK, Singh SM, Panda AK (2015). Protein recovery from inclusion bodies of *Escherichia coli* using mild solubilization process. Microb Cell Fact.

[24] Costa S, Almeida A, Castro A, Domingues L (2014). Fusion tags for protein solubility, purification and immunogenicity in *Escherichia coli*: the novel Fh8 system. Front Microbiol.

[25] Vaks L, Benhar I, Steinitz M (2014). Production of stabilized scFv antibody fragments in the *E. coli* bacterial cytoplasm. Human monoclonal antibodies: methods and protocols.

[26] Bach H, Mazor Y, Shaky S, Shoham-Lev A, Berdichevsky Y, Gutnick DL (2001). *Escherichia coli* maltose-binding protein as a molecular chaperone for recombinant intracellular cytoplasmic single-chain antibodies. J Mol Biol.

[27] Singh SM, Sharma A, Upadhyay AK, Singh A, Garg LC, Panda AK (2012). Solubilization of inclusion body proteins using *n*-propanol and its refolding into bioactive form. Protein Expr Purif.

[28] John RJS, Carpenter JF, Randolph TW. High pressure fosters protein refolding from aggregates at high concentrations. Proc Natl Acad Sci USA. 1999;96:13029–33. https://www.ncbi.nlm.nih.gov/pmc/articles/PMC23894/. Accessed 30 Aug 2018. 10.1073/pnas.96.23.13029 PMC23894 10557267

[29] Kudou M, Ejima D, Sato H, Yumioka R, Arakawa T, Tsumoto K (2011). Refolding single-chain antibody (scFv) using lauroyl-l-glutamate as a solubilization detergent and arginine as a refolding additive. Protein Expr Purif.

[30] Khan RH, Rao KB, Eshwari AN, Totey SM, Panda AK (1998). Solubilization of recombinant ovine growth hormone with retention of native-like secondary structure and its refolding from the inclusion bodies of *Escherichia coli*. Biotechnol Prog.

[31] Deng XK, Nesbit LA, Morrow KJ (2003). Recombinant single-chain variable fragment antibodies directed against *Clostridium difficile* toxin B produced by use of an optimized phage display system. Clin Diagn Lab Immunol.

[32] Cherrier MV, Kaufmann B, Nybakken GE, Lok S-M, Warren JT, Chen BR (2009). Structural basis for the preferential recognition of immature flaviviruses by a fusion-loop antibody. EMBO J.

[33] Baeshen NA, Baeshen MN, Sheikh A, Bora RS, Ahmed MMM, Ramadan HAI (2014). Cell factories for insulin production. Microb Cell Fact.

[34] da Costa SJM. Development of a novel fusion system for recombinant protein production and purification in *Escherichia coli.* 2013. http://repositorium.sdum.uminho.pt/handle/1822/24892. Accessed 29 Aug 2018.

[35] Dewi KS, Retnoningrum DS, Riani C, Fuad AM (2016). Construction and periplasmic expression of the anti-EGFRvIII scFv antibody gene in *Escherichia coli*. Sci Pharm.

[36] Kapust RB, Waugh DS (1999). *Escherichia coli* maltose-binding protein is uncommonly effective at promoting the solubility of polypeptides to which it is fused. Protein Sci Publ Protein Soc..

[37] Nallamsetty S, Waugh DS (2007). Mutations that alter the equilibrium between open and closed conformations of *Escherichia coli* maltose-binding protein impede its ability to enhance the solubility of passenger proteins. Biochem Biophys Res Commun.

[38] Sachdev D, Chirgwin JM (2000). Fusions to maltose-binding protein: control of folding and solubility in protein purification. Methods Enzymol.

[39] Kaplan W, Hüsler P, Klump H, Erhardt J, Sluis-Cremer N, Dirr H (1997). Conformational stability of pGEX-expressed *Schistosoma japonicum* glutathione *S*-transferase: a detoxification enzyme and fusion-protein affinity tag. Protein Sci Publ Protein Soc..

[40] Costa SJ, Almeida A, Castro A, Domingues L, Besir H (2013). The novel Fh8 and H fusion partners for soluble protein expression in *Escherichia coli*: a comparison with the traditional gene fusion technology. Appl Microbiol Biotechnol.

[41] Smith DB, Johnson KS (1988). Single-step purification of polypeptides expressed in *Escherichia coli* as fusions with glutathione *S*-transferase. Gene.

[42] Wiedemann C, Bellstedt P, Görlach M (2013). CAPITO—a web server-based analysis and plotting tool for circular dichroism data. Bioinformatics.

[43] Greenfield NJ (2006). Using circular dichroism collected as a function of temperature to determine the thermodynamics of protein unfolding and binding interactions. Nat Protoc.

[44] Uversky VN (2002). Natively unfolded proteins: a point where biology waits for physics. Protein Sci Publ Protein Soc..

[45] Malhotra A (2009). Tagging for protein expression. Methods Enzymol.

[46] Young CL, Britton ZT, Robinson AS (2012). Recombinant protein expression and purification: a comprehensive review of affinity tags and microbial applications. Biotechnol J.

