# Supplementary figures and images for: Evaluation of scFv protein recovery from E. coli by in vitro refolding and mild solubilization process
Source: Microb Cell Fact. 2019 Jan 14;18:5. doi: 10.1186/s12934-019-1053-9 (PMC6330739; doi:10.1186/s12934-019-1053-9)

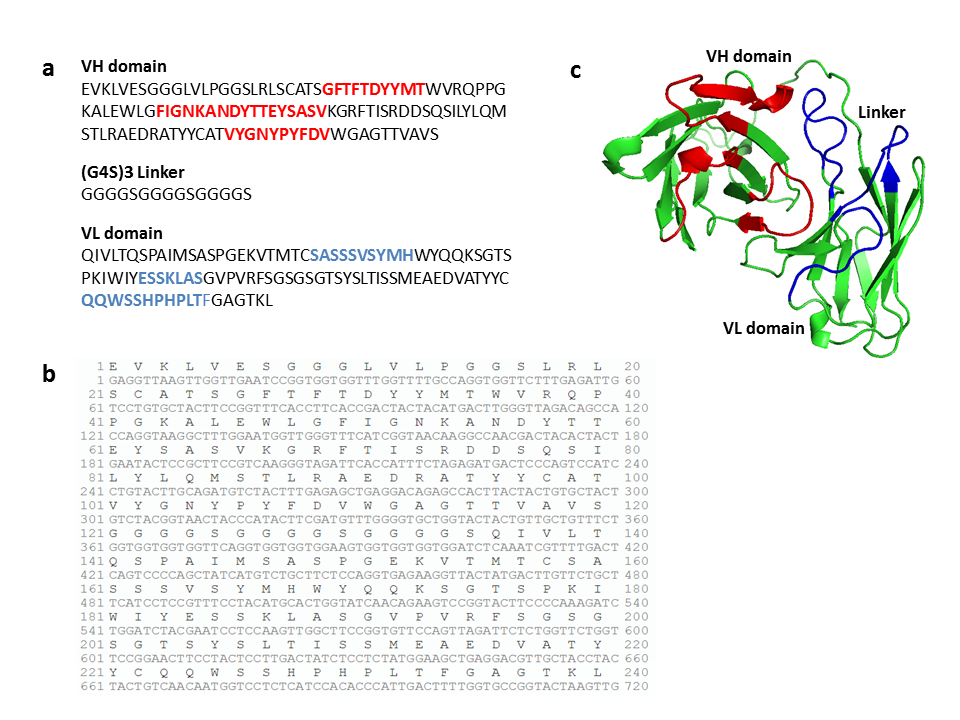

Supplement: Supplementary file 1 — Additional file 1: Figure S1. Construction of scFv antibody gene. (a) The amino acid sequences of variable heavy chain (VH) and the variable light chain (VL) were joined with most commonly used peptide linker (G4S)3 sequence. Red and blue color residues are denoting CDRs of VH and VL respectively. (b) The newly designed scFv protein sequence was reverse translated in to DNA sequence by using computational tool. (c) Homology model of scFv protein molecule was designed by Swiss PDB viewer. [file 12934_2019_1053_MOESM1_ESM.png]

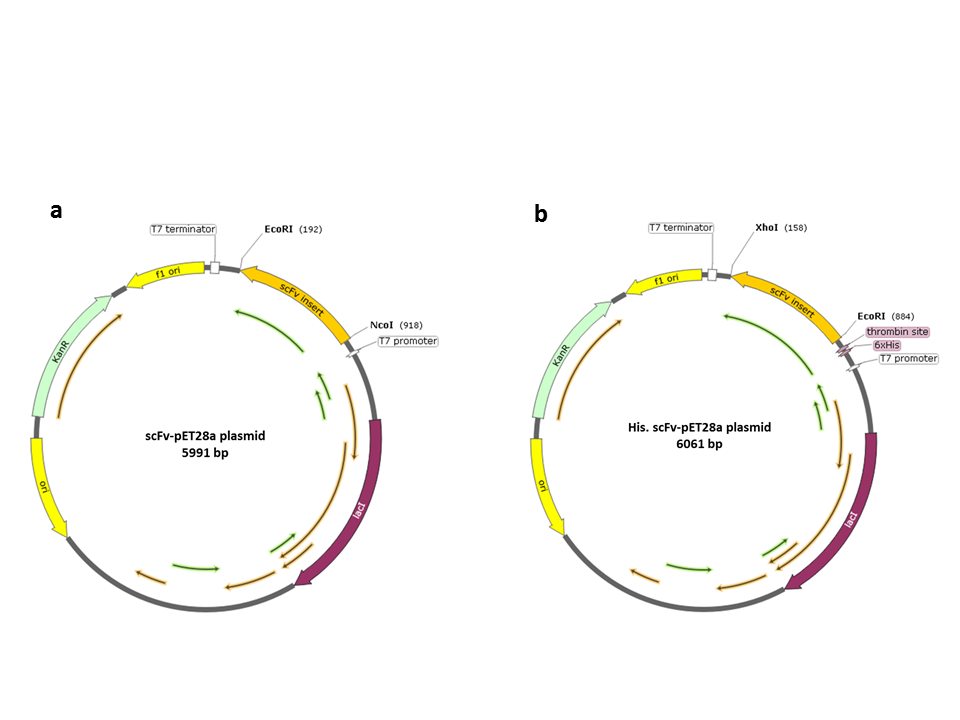

Supplement: Supplementary file 2 — Additional file 2: Figure S2. Complete map of scFv-pET28a and His.scFv-pET28a plasmid. The scFv gene was cloned (a) using NcoI and EcoRI retriction sites of pET28a(+) to skip 6xHis tag and (b) using EcoRI and XhoI restriction sites of pET28a(+) to incorporate 6xHis tag during expression. The complete recombined map was created with SnapGene. [file 12934_2019_1053_MOESM2_ESM.png]

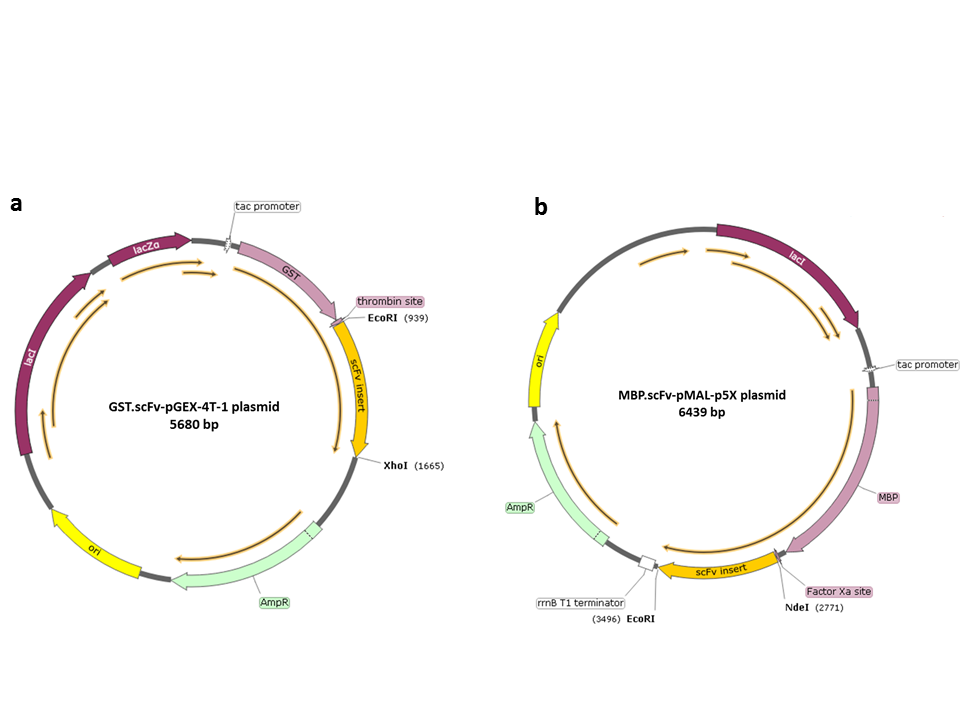

Supplement: Supplementary file 3 — Additional file 3: Figure S3. Complete map of GST.scFv-pGEX-4T-1 and MBP.scFv-pMAL-p5X plasmid. The scFv gene was inserted (a) using EcoRI and XhoI restriction sites in pGEX-4T-1 expression vector and (b) using NdeI and EcoRI restriction sites in pMAL-p5X expression vector in order to express fusion scFv protein with GST and MBP tag respectively. The recombination was created with SnapGene. [file 12934_2019_1053_MOESM3_ESM.png]

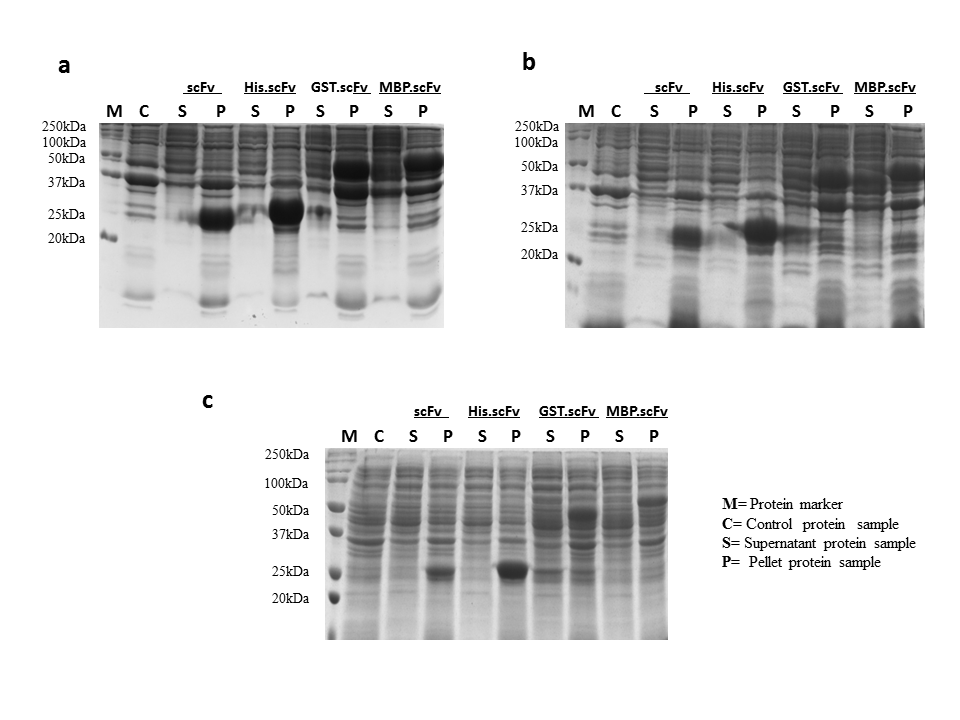

Supplement: Supplementary file 4 — Additional file 4: Figure S4. Expression pattern of scFv antibody protein without and with different fusion tags at different biological conditions. All of the four clones, scFv-pET28a(+), His.scFv-pET28a(+), GST.scFv-pGEX-4T-1 and MBP.scFv-pMAL-p5X were expressed in (a) 1 mM IPTG concentration at 37 °C for 4 h, then (b) 0.5 mM IPTG concentration at 30 °C for 4 h and (c) in 0.5 mM IPTG concentration at 20 °C for overnight. [file 12934_2019_1053_MOESM4_ESM.png]

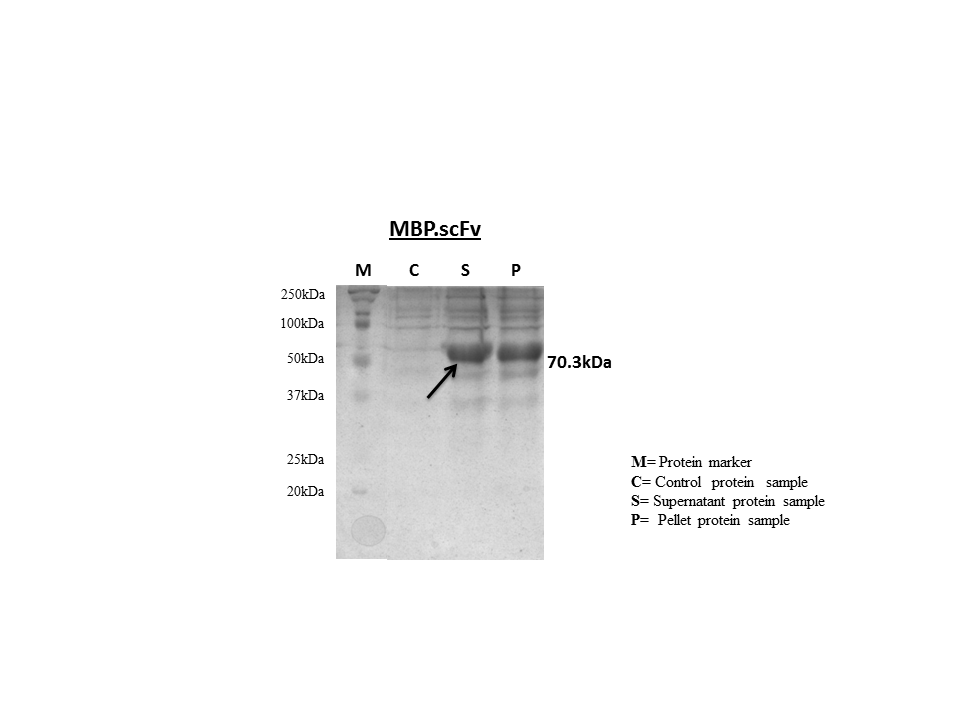

Supplement: Supplementary file 5 — Additional file 5: Figure S5. Periplasmic expression pattern of scFv antibody protein in fusion with MBP tag. MBP.scFv-pMAL-p5X recombinant plasmid was used to transform E. coli BL21 bacteria and cells were grown up to OD (A600 ~ 0.5) followed by expression with 0.3 mM IPTG concentration for 2 h at 37 °C. Harvested cells were used for protein extraction following periplasmic extraction method. The protein isolated from both in supernatant (S) and pellet (P) was used to separate by SDS 12 % polyacrylamide gel electrophoresis. Un-induced transformed cells were also extracted by periplasmic extraction method and also allowed for SDS 12 % polyacrylamide gel electrophoresis as control (C) sample. [file 12934_2019_1053_MOESM5_ESM.png]

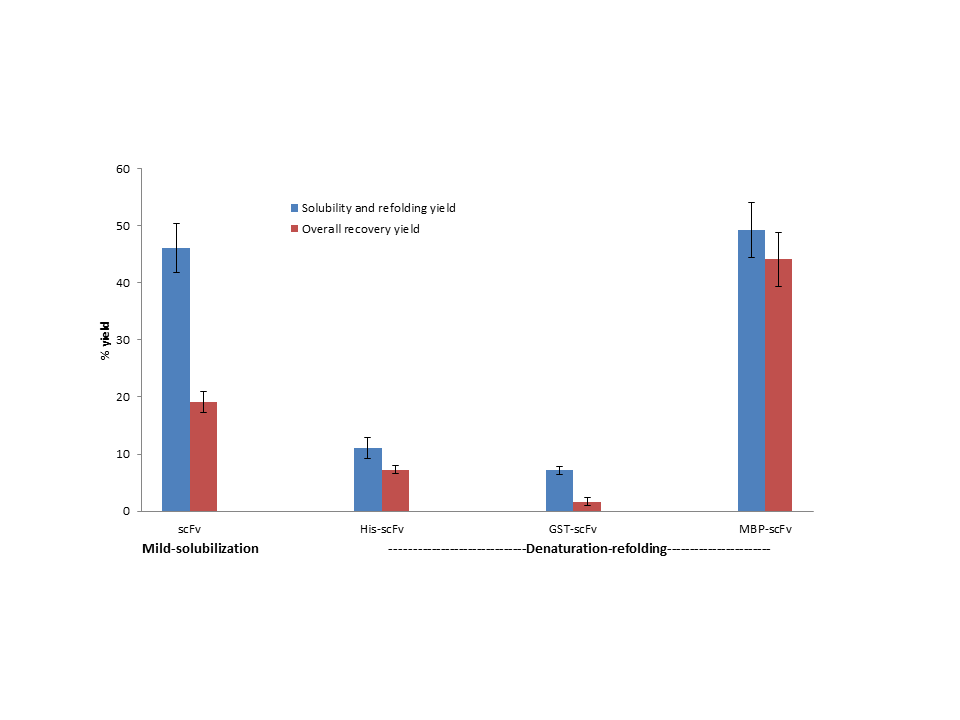

Supplement: Supplementary file 6 — Additional file 6: Figure S6. Percent (%) study of solubility, refolding and overall recovery yield of scFv protein. The IBs of scFv were solubilized mildly and strongly with the corresponding mild and strong denaturing agents. Completely denatured scFvs fusion with 6xHis, GST and MBP tags were further allowed for in vitro refolding. The recovery yields of soluble and refolded scFv proteins achieved by mild solubilization and denaturation-refolding method were converted into percent yields as per the quantity of initial pellet protein. Furthermore, overall percent yields of pure scFvs recovered by these two methods were calculated and compared with each other. [file 12934_2019_1053_MOESM6_ESM.png]
