# Supplementary material for: Evaluation of scFv protein recovery from E. coli by in vitro refolding and mild solubilization process
Source: Microb Cell Fact. 2019 Jan 14;18:5. doi: 10.1186/s12934-019-1053-9 (PMC6330739; doi:10.1186/s12934-019-1053-9)
Supplement: Supplementary file 7 — Additional file 7. Primer sequences for cloning scFv synthetic gene without and with His, GST and MBP fusion tags. [file 12934_2019_1053_MOESM7_ESM.docx]

**Additional file 7:** Primer sequences for cloning scFv synthetic gene without and with His, GST and MBP fusion tags.

1. Primers for scFv cloning in pET28a(+) expression vector skipping His tag

*NcoI*

Forward: 5' CGC CAT ATG GAG GTT AAG TTG GTT GAA TCC 3'

*EcoRI*

Reverse: 5' CGG AAT TCC AAC TTA GTA CCG GCA C 3'

1. Primers for scFv cloning in pET28a(+) expression vector with N-terminal His tag

*XhoI*

Forward: 5' CGC CTC GAG GAG GTT AAG TTG GTT GAA TCC 3'

*EcoRI*

Reverse: 5' CGG AAT TCC AAC TTA GTA CCG GCA C 3'

1. Primers for scFv cloning in pGEX-4T-1 expression vector with N-terminal GST tag

*XhoI*

Forward: 5' CGC CTC GAG GAG GTT AAG TTG GTT GAA TCC 3'

*EcoRI*

Reverse: 5' CGG AAT TCC AAC TTA GTA CCG GCA C 3'

1. Primers for scFv cloning in pMAL-p5X expression vector with N-terminal MBP tag

*NdeI*

Forward: 5' CGC CAT ATG GAG GTT AAG TTG GTT GAA TCC 3'

*EcoRI*

Reverse: 5' CGG AAT TCC AAC TTA GTA CCG GCA C 3'
